# Metabolomic biomarkers for the diagnosis and post-transplant outcomes of AFP negative hepatocellular carcinoma

**DOI:** 10.3389/fonc.2023.1072775

**Published:** 2023-02-09

**Authors:** Zuyuan Lin, Huigang Li, Chiyu He, Modan Yang, Hao Chen, Xinyu Yang, Jianyong Zhuo, Wei Shen, Zhihang Hu, Linhui Pan, Xuyong Wei, Di Lu, Shusen Zheng, Xiao Xu

**Affiliations:** ^1^ Department of Hepatobiliary and Pancreatic Surgery, Affiliated Hangzhou First People’s Hospital, Zhejiang University School of Medicine, Hangzhou, China; ^2^ The First Affiliated Hospital, Zhejiang University School of Medicine, Hangzhou, China; ^3^ Key Laboratory of Integrated Oncology and Intelligent Medicine of Zhejiang Province, Affiliated Hangzhou First People’s Hospital, Zhejiang University School of Medicine, Hangzhou, China; ^4^ National Health Commission Key Laboratory of Combined Multi-organ Transplantation, Hangzhou, China; ^5^ Institute of Organ Transplantation, Zhejiang University, Hangzhou, China; ^6^ Department of Hepatobiliary and Pancreatic Surgery, Shulan (Hangzhou) Hospital, Zhejiang Shuren University School of Medicine, Hangzhou, China; ^7^ Zhejiang University School of Medicine, Hangzhou, China

**Keywords:** hepatocellular carcinoma, cirrhosis, AFP, metabolomics, nomogram

## Abstract

**Background:**

Early diagnosis for α-fetoprotein (AFP) negative hepatocellular carcinoma (HCC) remains a critical problem. Metabolomics is prevalently involved in the identification of novel biomarkers. This study aims to identify new and effective markers for AFP negative HCC.

**Methods:**

In total, 147 patients undergoing liver transplantation were enrolled from our hospital, including liver cirrhosis patients (LC, n=25), AFP negative HCC patients (NEG, n=44) and HCC patients with AFP over 20 ng/mL (POS, n=78). 52 Healthy volunteers (HC) were also recruited in this study. Metabolomic profiling was performed on the plasma of those patients and healthy volunteers to select candidate metabolomic biomarkers. A novel diagnostic model for AFP negative HCC was established based on Random forest analysis, and prognostic biomarkers were also identified.

**Results:**

15 differential metabolites were identified being able to distinguish NEG group from both LC and HC group. Random forest analysis and subsequent Logistic regression analysis showed that PC(16:0/16:0), PC(18:2/18:2) and SM(d18:1/18:1) are independent risk factor for AFP negative HCC. A three-marker model of Metabolites-Score was established for the diagnosis of AFP negative HCC patients with an area under the time-dependent receiver operating characteristic curve (AUROC) of 0.913, and a nomogram was then established as well. When the cut-off value of the score was set at 1.2895, the sensitivity and specificity for the model were 0.727 and 0.92, respectively. This model was also applicable to distinguish HCC from cirrhosis. Notably, the Metabolites-Score was not correlated to tumor or body nutrition parameters, but difference of the score was statistically significant between different neutrophil-lymphocyte ratio (NLR) groups (≤5 vs. >5, P=0.012). Moreover, MG(18:2/0:0/0:0) was the only prognostic biomarker among 15 metabolites, which is significantly associated with tumor-free survival of AFP negative HCC patients (HR=1.160, 95%CI 1.012-1.330, P=0.033).

**Conclusion:**

The established three-marker model and nomogram based on metabolomic profiling can be potential non-invasive tool for the diagnosis of AFP negative HCC. The level of MG(18:2/0:0/0:0) exhibits good prognosis prediction performance for AFP negative HCC.

## 1 Introduction

Liver cancer ranks the 6th most prevalent cancer, and the related mortality ranks the 4th (1). Hepatocellular carcinoma (HCC) comprises around 80% of all the liver cancer cases. China has the heaviest HCC burden worldwide owing to the prevalence of Hepatitis B. HCC is characterized by insidious onset and rapid progress, and prone to metastasis (2). Therefore, many HCC patients are no longer suitable for surgical treatment when they are diagnosed. Most HCC evolves from liver cirrhosis (3). Distinguishing HCC from liver cirrhosis, especially in the early stage, is conducive to clinical decision-making and thus improves the prognosis. α-fetoprotein (AFP) is the most widely used serologic marker for the HCC diagnosis. However, its diagnostic power has been continuously challenged, because up to 50% of small HCC do not secrete AFP and it is elevated in only 20% of early stage HCC patients (4). Moreover, AFP may also deviate from normal value in cirrhosis or hepatitis patients (5). Therefore, the exploration for novel and effective biomarkers for AFP negative HCC is critically important.

Metabolomics is a high throughput and quantitative approach to measure the low-molecular-weight metabolites under specific conditions (6). It is capable of detecting metabolic changes in different pathological or physiological status, which has been an effective tool in disease diagnosis, mechanism study and drug screening (7). Currently, it has shown great promise as a means to identify new biomarkers for various types of cancer, including HCC (8). Acetylcarnitine was identified by metabolomic profiling as a serum diagnostic marker for HCC (9). Liu et al. identified 32 metabolites by metabolomics that altered between HCC and liver cirrhosis (LC), and achieve 100% sensitivity with these markers (10). Wu et al. even established a diagnostic model for HCC from LC based on GC/MS in urine sample (11). However, metabolomic profiling specific for AFP negative HCC is still needed to improve the diagnostic accuracy for HCC. In this study, we enrolled patients of different status related to HCC. By comparing the metabolomic profiling between groups, we successfully identified metabolites capable of screening out AFP negative HCC and further established a novel model.

## 2 Materials and methods

### 2.1 Study population and data collection

25 liver cirrhotic patients (LC group) and 122 HCC patients including 44 AFP negative HCC patients (NEG group), 78 HCC patients with AFP over 20 ng/ml (POS group) in the First Affiliated Hospital of Zhejiang University School of Medicine from April 2012 to December 2016 were enrolled in the study. All Patients in the LC and HCC group underwent liver transplantation and were diagnosed according to post-transplant pathological examination. The exclusion criteria included patients younger than 18 years, undergoing multiorgan transplantation or re-transplantation, or with missing essential data for analysis. Another cohort of 52 healthy control samples (HC group) collected from the same batch of individuals who underwent healthy examination. We collected the data including demographics, body mass index (BMI), pre- operative AFP level, alanine transaminase (ALT) level, aspartate transaminase (AST) level, morphological features (tumor number and largest tumor size), skeletal muscle index [SMI, to define sarcopenia (12)], neutrophil-lymphocyte ratio (NLR), post-transplant recurrence, and patients’ survival for analysis. Informed consent was obtained from all the participants, and the study protocol was approved by the Human Ethics Committee of the hospital.

### 2.2 Sample preparation

Peripheral blood samples (EDTA-K2 anticoagulant) were collected from fasted patients or healthy volunteers in the morning of LT or healthy examination, and centrifuged at 3000 rpm for 10 min, then stored the plasma at −80°C, until use. The plasma samples were thawed at 4°C, and the quality control (QC) samples were prepared by pooling aliquots (10 μl) of each sample. Acetonitrile (800 μl) was added to the plasma (200 μl) sample and vortexed for 1 min. We then incubated the mixture at room temperature for 1 min and centrifuged it at 14000 rpm for 10 min at 4°C. The acquired clear supernatant was transferred to UPLC vials, and was then stored at 4°C until detection. The pretreatment of the QC samples was the same as that for the test samples.

### 2.3 UPLC–MS analysis of samples

We performed reversed-phase analysis on a Waters ACQUITY Ultra Performance LC system using an ACQUITY UPLC BEH C18 analytical column (i.d., 2.1 mm × 100 mm; particle size 1.7 mm; pore size, 130 Å). We then used water/formic acid (99.9:0.1 v/v) as mobile phase A and acetonitrile/formic acid (99.9:0.1 v/v) as mobile phase B. A linear gradient LC system (Waters, Milford MA) was optimized as follows: the composition of mobile phase B was changed from 3% to 80% in 7 min, reached 98% in 8 min and held for 5 min, and then reached 100% in 1** **min and held for 3 min. The sample manager was kept at 4**°**C, with an injection volume of 2 μl for each analysis. The QC samples were injected at regular intervals (every 14 samples) throughout the analytical run. These inserted QC samples were used to evaluate the repeatability of sample pretreatment and monitor the stability of the LC–MS system during sequence analysis.

We used a Waters Q-TOF Premier mass spectrometer to perform the mass spectrometry in positive ion electrospray mode. The instrumental parameters were set as follows: The mass scan range was 50 m/z–1000 m/z using an accumulation time of 0.2 s per spectrum; the MS acquisition rate was set to 0.3 s with a 0.02 s inter scan delay; high-purity nitrogen was used as nebulizer and drying gas. The nitrogen drying gas was at a constant flow rate of 600 L/h, and the source temperature was set at 120**°**C. For the positive mode, the capillary voltage was set at 3.0 kV and the sampling cone voltage was set at 45.0 V. Argon was used as collision gas. MS/MS analysis was performed on the mass spectrometer set at different collision energies of 10 eV–50 eV according to the stability of each metabolite. The time of flight analyzer was used in V mode and tuned for maximum resolution (>10,000 resolving power at m/z 556.2771). The instrument was previously calibrated with sodium formate; the lock mass spray for precise mass determination was set by leucine enkephalin at 556.2771 m/z with concentration of 0.5 ng/L in the positive ion mode. All analyses were acquired using the lock spray to ensure accuracy and reproducibility.

### 2.4 Data processing and statistical analysis

We referred to our previously published metabolomic data (13). The dataset was generated based on the retention time, m/z, and normalized signal intensity of the peaks. The preprocessed data obtained by MassLynx were exported and analyzed using SIMCA-P 14.1 (Umetrics AB, Sweden). Firstly, principal component analysis (PCA) was introduced to evaluate the reliability of the resulting dataset (including QC samples). Secondly, supervised orthogonal partial least squares discriminant analysis (OPLS-DA) was performed to better distinguishing the two groups. Potential biomarkers of differentiating AFP negative HCC patients from LC and HC groups were selected according to the Variable Importance in the Projection (VIP) values, fold change (FC), and Wilcoxon Test. Statistical analysis including logistic regression and cox regression was performed using SPSS version 25.0 statistical software (SPSS inc. Chicago, IL, USA) and GraphPad Prism version 9 (GraphPad, La Jolla, CA, USA). Random forest analysis and nomogram construction were performed by R Version 3.6.1. Area under the time-dependent receiver operating characteristic curve (AUROC) were used to evaluate discriminative ability. The AUROC difference is performed using DeLong’s test. The Hosmer-Lemeshow (HL) goodness-of-fit test was used to assess the calibration of the model. Mann-Whitney U test were used to compare the Metabolite-Score between different groups. Kaplan-Meier analysis and Breslow test were used to compare the survival between groups. P < 0.05 was considered statistically significant throughout the study.

## 3 Results

### 3.1 Baseline characteristics

147 patients included in this study underwent LT for HCC or cirrhosis treatment, and 52 healthy volunteers were also enrolled. Of all the patients with different liver diseases, 132 were male (89.8%) and 15 were female (10.2%), while 14 were male (26.9%) and 38 were female (73.1%) in healthy controls. The mean age in LC group and NEG group was 47.9 ± 9.8 and 53.2 ± 8.8 years, respectively (P=0.021). This could be explained by the fact that cirrhosis is an intermediate process of chronic hepatic disease developing to HCC. The AFP level in these two groups was 79.9 ± 275.4 and 8.4 ± 5.5 ng/mL, respectively (P=0.170). The difference of liver functions (including ALT and AST) was not significant between the two groups. Baseline features of all the study subjects including HC group and POS group were listed in **Table 1**, and particular features of tumor patients are listed in **Supplementary Table S1**.

**Table 1:** Baseline characteristics of patients with liver disease.

	Liver cirrhosis (n=25)	AFP negative HCC (n=44)	AFP positive HCC (n=78)	Healthy controls (n=52)	P value*
Age (years)	47.9 ± 9.8	53.2 ± 8.8	51.6 ± 8.1	37.8 ± 10.4	0.021
Male gender, n (%)	22 (88.0)	40 (90.9)	70 (89.7)	14 (26.9)	0.700
AFP (ng/mL)	79.9 ± 275.4	8.4 ± 5.5	7091.4 ± 15697.9	6.3 ± 23.4^#^	0.170
ALT (U/L)	123.7 ± 197.6	64.1 ± 101.9	50.7 ± 54.8	17.6 ± 12.4	0.836
AST (U/L)	132.8 ± 249.3	94.9 ± 241.1	69.7 ± 62.5	20.4 ± 7.0	0.400

*P: Liver cirrhosis vs. AFP negative HCC group.

^#^: There was one case of missing data.

AFP, α-fetoprotein; HCC, hepatocellular carcinoma; ALT, alanine aminotransferase; AST, aspartate aminotransferase.

### 3.2 Metabolomic markers for AFP negative HCC

**Figure 1:**
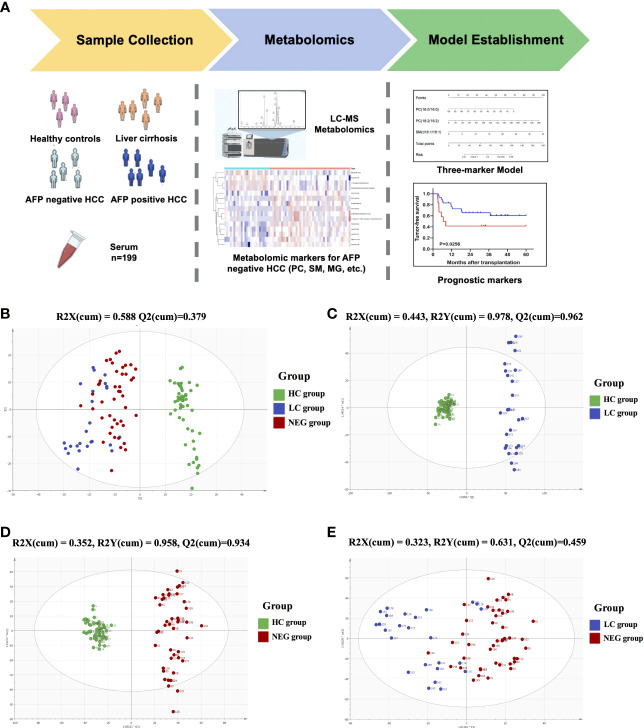
The metabolomics profiling for plasma samples. **(A)** General workflow for this study. **(B)** PCA score plot for 52 healthy controls, 25 liver cirrhosis patients, 122 HCC patients and 15 quality controls. **(C)** OPLS-DA score plot for HC and LC group. **(D)** OPLS-DA score plot for HC and NEG group. **(E)** OPLS-DA score plot for LC and NEG group.

Metabolomic profiling was performed on the plasma of 52 healthy volunteers and 147 patients with HCC or cirrhosis, and general workflow of this study is listed as **Figure 1A**. The total ion chromatograms of a single sample from each group were acquired by the UPLC-MS platform. Using MZmine ver. 2.0 software, this pre-treatment revealed 1242 integral peaks following extraction ion chromatography detection in all samples, which was reported in our previous work (13). PCA plot (R2X=0.631, Q2 = 0.421) showed that QC sample cluster together, indicating the high stability and reproducibility of the instrument (**Supplementary Figure S1**). Besides, HC group showed an obvious separation from NEG HCC group and LC group, while NEG HCC group was roughly separated from LC group (**Figure 1B**).

**Figure 2:**
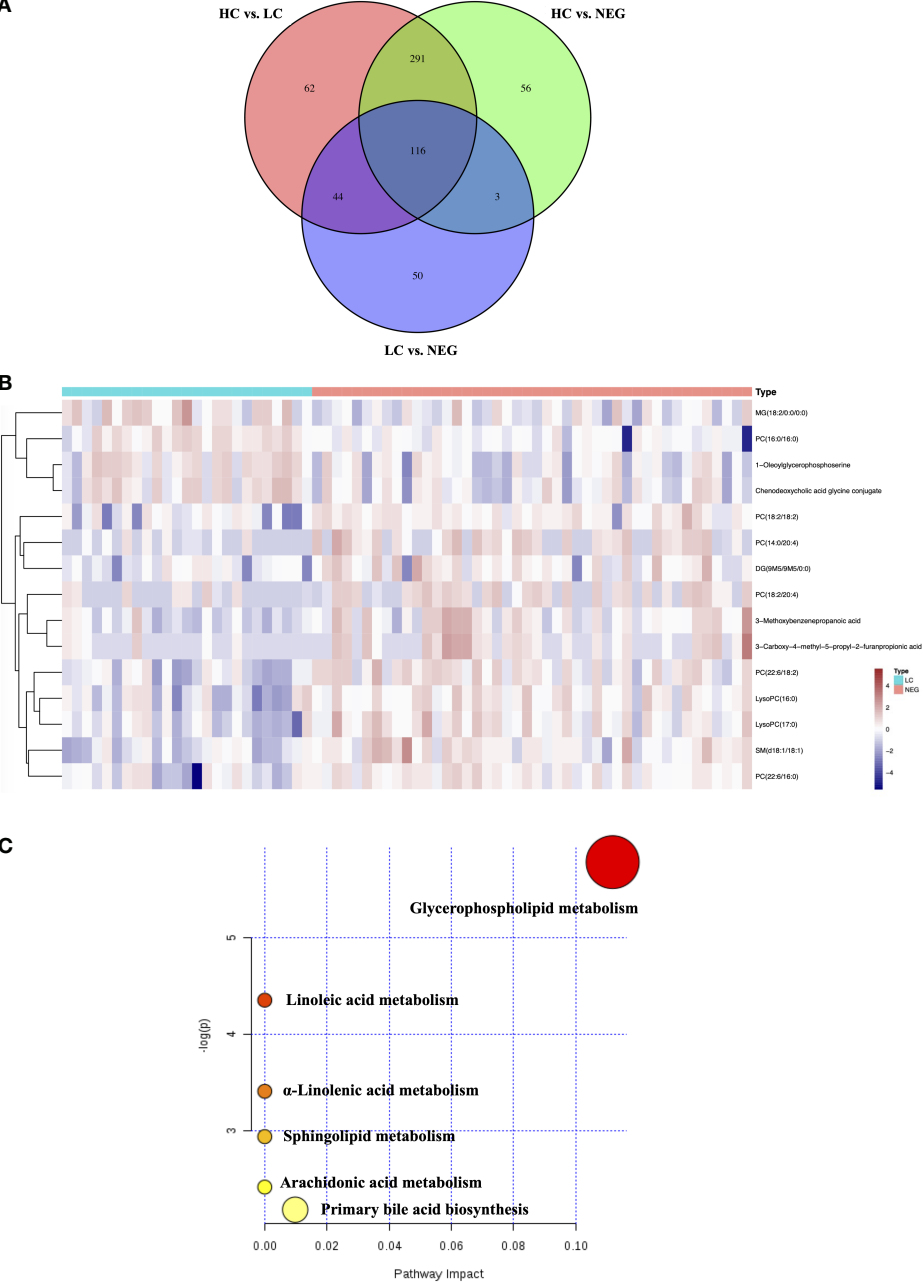
Differential metabolites for AFP negative HCC. **(A)** Venn diagram of the differential ions in HC vs. LC, HC vs. NEG and LC vs. NEG. **(B)** Heatmap of 15 differentially expressed metabolites between LC and NEG group according to the normalized intensity. **(C)** Summary of altered pathways AFP negative HCC patients compared to liver cirrhosis patients, as analyzed by MetaboAnalyst platform (https://www.metaboanalyst.ca/). .

In order to identify metabolomic markers for AFP negative HCC, pair-wise comparisons were performed among HC, LC and NEG group based on OPLS-DA models (**Figures 1C–E**) and Wilcoxon Test. Validation of the OPLS-DA model of LC and NEG group was obtained from 200 permutation tests (**Supplementary Figures S2A–C**). The validation plot demonstrated that the original model was valid: the Q2 regression line had a negative intercept, and the intercepts of R2 were lower than the original point to the right. S-plots of these OPLS-DA models were further investigated to acquire the correlation value of metabolites (**Supplementary Figures S2D–F**). Ions with a variable importance value (VIP) >1, fold change (FC) >1.5 and P<0.01 were selected. Thus, 116 overlapping ions were selected for further identification (**Figure 2A**). By excluding those ions with over one third cases of ‘0’ value, 15 metabolites including MG (monoacylglyceride), PC (phosphatidylcholine), DG (diglyceride) and SM (sphingomyelin) were finally selected (**Table 2** and **Supplementary Table S2**), and their VIP values and correlation values were also listed. In comparison to LC group, 4 metabolites (Chenodeoxycholic acid glycine conjugate, MG(18:2/0:0/0:0), 1-Oleoylglycerophosphoserine, PC(16:0/16:0)) were significantly decreased in NEG group, whereas 11 metabolites (DG(9M5/9M5/0:0), PC(22:6/16:0), SM(d18:1/18:1), LysoPC(17:0), LysoPC(16:0), PC(22:6/18:2), PC(18:2/18:2), 3-Methoxybenzenepropanoic acid, PC(18:2/20:4), 3-Carboxy-4-methyl-5-propyl-2-furanpropionic acid, PC(14:0/20:4)) were significantly elevated (**Figure 2B**).

**Table 2:** Differential ions and referred metabolites between LC and NEG group.

Ions	Mean (LC)	Mean (NEG)	logFC	P value	VIP value	Correlation value	Metabolites
var297	97.351	44.007	-1.145	3.38E-03	1.336	-0.631	Chenodeoxycholic acid glycine conjugate
var499	7.985	3.980	-1.004	1.15E-03	1.468	-0.383	MG(18:2/0:0/0:0)
var634	10.424	5.654	-0.883	1.93E-04	1.468	-0.677	1-Oleoylglycerophosphoserine
var690	60.707	34.012	-0.836	1.08E-05	1.772	-0.783	PC(16:0/16:0)
var350	1.321	2.069	0.648	2.46E-03	1.226	0.335	DG(9M5/9M5/0:0)
var265	69.075	111.190	0.687	5.81E-04	1.413	0.577	PC(22:6/16:0)
var61	7.749	12.562	0.697	6.87E-06	1.419	0.473	SM(d18:1/18:1)
var380	2.362	4.057	0.780	5.08E-04	1.387	0.557	LysoPC(17:0)
var4	31.630	57.377	0.859	8.63E-04	1.347	0.528	LysoPC(16:0)
var169	3.388	6.484	0.937	9.04E-06	1.545	0.574	PC(22:6/18:2)
var325	24.917	51.680	1.052	1.94E-04	1.333	0.436	PC(18:2/18:2)
var312	2.192	6.600	1.590	1.76E-04	1.184	0.346	3-Methoxybenzenepropanoic acid
var905	3.500	11.605	1.729	9.60E-04	1.191	0.472	PC(18:2/20:4)
var898	0.150	0.645	2.107	2.50E-03	1.089	0.378	3-Carboxy-4-methyl-5-propyl-2-furanpropionic acid
var810	0.441	1.942	2.140	2.35E-04	1.360	0.547	PC(14:0/20:4)

FC, fold change; VIP, Variable Importance in the Projection; MG, monoacylglyceride; PC, phosphatidylcholine; DG: diglyceride; 9M5, 9-(3-methyl-5-pentylfuran-2-yl)nonanoic acid; SM, sphingomyelin.

The biological pathways involved in the metabolism of these 15 differential metabolites were determined by enrichment analysis using MetaboAnalyst. All matched pathways were shown according to p values from the pathway enrichment analysis (y-axis) and pathway impact values from pathway topology analysis (x-axis) (14), with the most impacted pathways colored in red. One pathway was considered specifically related to AFP negative HCC, that is, glycerophospholipid metabolism (**Figure 2C**).

### 3.3 A novel model for the diagnosis of AFP negative HCC

**Figure 3:**
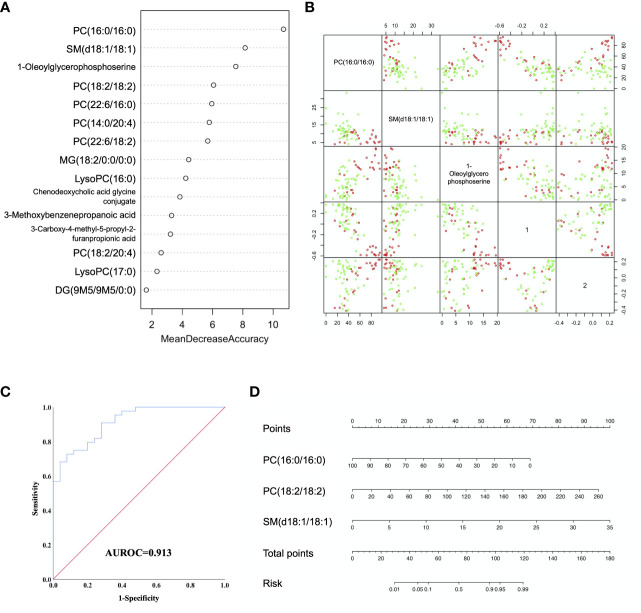
Diagnostic model for AFP negative HCC based on Random Forest (RF) analysis. **(A)** MDA plot of 15 differentially expressed metabolites based on RF analysis between LC and NEG group. **(B)** Predictors and PCoA plot based on RF analysis, and Scatter plots showing correlation distribution between each feature and PCoA1/2 axes. **(C)** ROC curve showing the ability of three-marker model to distinguish AFP negative HCC patients from liver cirrhosis patients. **(D)** Diagnostic nomogram for AFP negative HCC based on the three-marker model.

Random forest (RF) analysis was further used to discriminate AFP negative HCC patient from liver cirrhosis patients based on 15-metabolites panel, which showed relatively low error rate of 25.49% in the training set. Moreover, the prediction of validation data based on training set RF models also yielded satisfactory results with error rate of 14.28% for LC vs. NEG. In order to identify potential biomarkers for AFP negative HCC, the top 7 ranked differential metabolites in the respective models were selected according to the mean decrease accuracy (MDA), which denoted the percent decrease in accuracy when the trial was performed in the absence of the metabolite (**Figure 3A**). The PCoA plot also showed these two groups of samples could almost cluster separately (**Figure 3B**). Subsequent Logistic regression analysis showed that PC(16:0/16:0), PC(18:2/18:2) and SM(d18:1/18:1) were independent risk factors distinguishing AFP negative HCC from liver cirrhosis patients (**Supplementary Table S3**). Thus, a three-marker model was constructed: Metabolites-Score = -0.071* PC(16:0/16:0) + 0.038* PC(18:2/18:2) + 0.293* SM(d18:1/18:1)-0.553.The ROC curve for the three-marker model was then constructed and a nomogram was then established as well (**Figures 3C, D**). The model showed good discrimination (AUROC=0.913, 95%CI 0.848-0.977, P<0.001) and calibration (HL P=0.739). According to the model, AFP negative patients but with Metabolites-Score more than 1.2895 could be regarded as having a high risk of HCC. The sensitivity and specificity for the model were 0.727 and 0.92, respectively.

### 3.4 Model for the diagnosis of HCC

**Figure 4:**
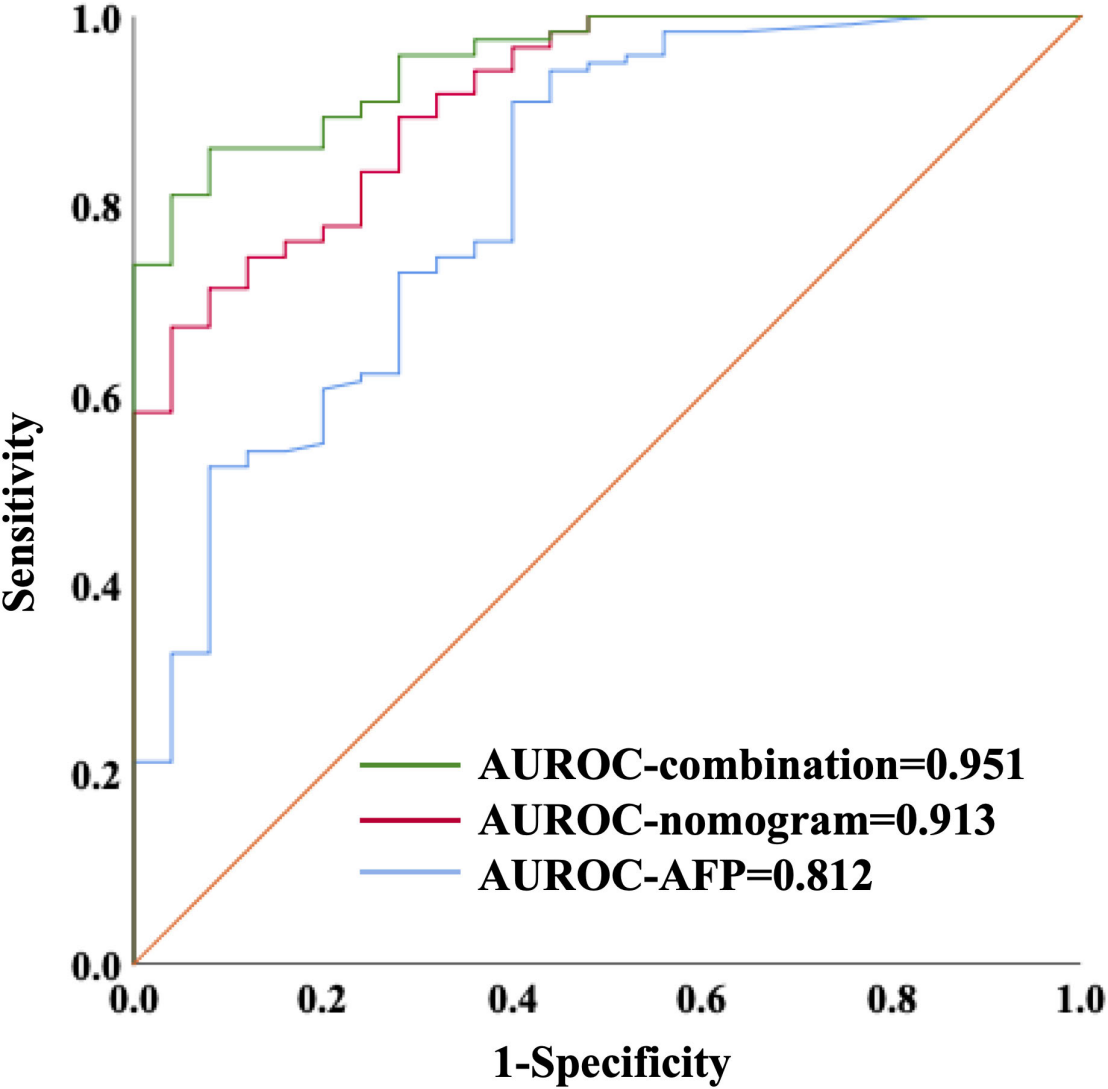
ROC curves showing diagnostic value of nomogram combining with AFP in distinguishing HCC patients from liver cirrhosis patients.

We further validated our three-marker model in all patients with HCC or cirrhosis. The diagnostic value of this model was assessed, showing a good discrimination (AUROC=0.912, 95%CI 0.857-0.967, P<0.001) and calibration (HL P=0.645). The cut-off value of Metabolites-Score was also set at 1.2895 with a sensitivity of 0.713 and a specificity of 0.92. To compare the diagnostic performance between our model and AFP, we also performed ROC analysis for AFP and the AUROC was 0.812 (95%CI 0.716-0.909, P<0.001). When the cut-off value was set at 3.7 ng/ml, the sensitivity and specificity were 0.91 and 0.6, respectively. Though the AUROC of our three-marker model was higher than that of AFP, the difference was not significant between them (ΔAUROC=0.1, P=0.13). By combining AFP with three-marker model, we are able to achieve a higher accuracy for diagnosis with an AUROC of 0.951 (95%CI 0.917-0.986, P<0.001, **Figure 4**) and a HL P value of 0.216. The diagnostic performance of the combination model was significantly better than three-marker model (ΔAUROC=0.039, P=0.006) or AFP along (ΔAUROC=0.139, P=0.014), with a positive predictive value of 0.981 and a negative predictive value of 0.575 (**Supplementary Table S4**).

### 3.5 Correlation between the metabolites-score and clinical parameters

**Figure 5:**
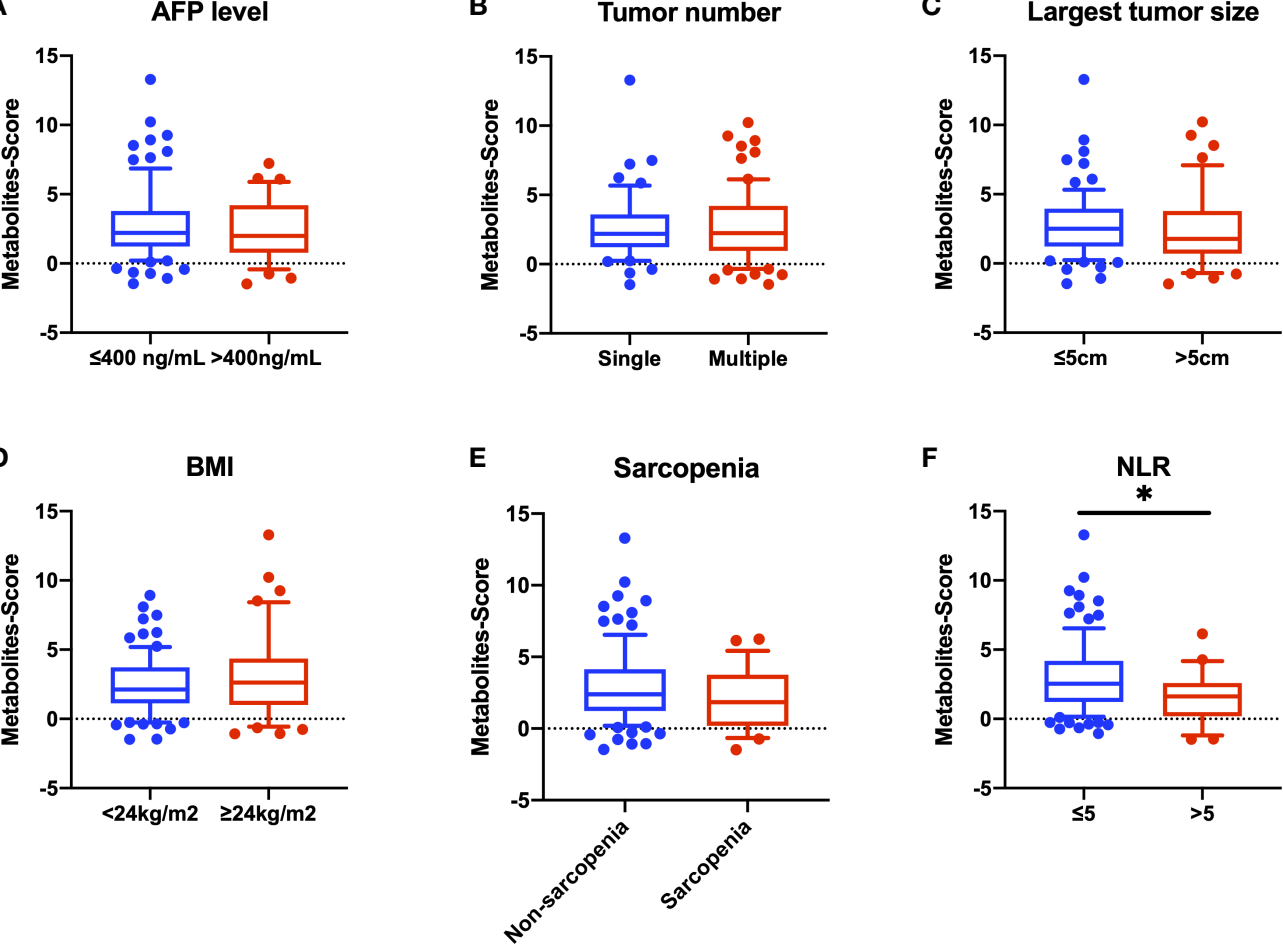
Comparison of Metabolites-Score in different groups divided by clinical parameters. Metabolites-Score showed no significant difference in groups divided by tumor parameters and body nutrition parameters, but was significantly correlated to NLR level. **(A)** Bar plot for AFP ≤ 400 ng/mL group vs. >400ng/mL group. **(B)** Bar plot for single tumor group vs. multiple tumor group. **(C)** Bar plot for largest tumor size ≤ 5 cm group vs. >5 cm group. **(D)** Bar plot for BMI<24kg/m2 group vs. ≥24kg/m2 group. **(E)** Bar plot for non-sarcopenia group vs. sarcopenia group. **(F)** Bar plot for NLR ≤ 5 group vs. >5 group. Data are expressed as median (10-90 percentile range) (*P < 0.05, Mann–Whitney U test).

We further explore the relationship between Metabolites-Score and clinical parameters, and 122 HCC patients were enrolled. We stratified all HCC patients into two groups according to AFP level (≤400 ng/mL and >400ng/mL), tumor number (single and multiple) and largest tumor size (≤5cm and >5cm), though no statistically significant difference in Metabolites-Score were found between any two groups (P>0.05, **Figures 5A–C**). In addition, we analyze the relationship between Metabolites-Score and body nutrition status in all HCC patients. In overweight patients group (BMI≥24kg/m^2^), the Metabolites-Score was higher than that in normal weight patients group (BMI<24kg/m^2^, 3.81 ± 3.13 vs. 2.99 ± 2.13, P=0.243, **Figure 5D**). In sarcopenic patient group, the Metabolites-Score was lower than that in non-sarcopenic patient group (2.56 ± 2.11 vs. 3.46 ± 2.60, P=0.155, **Figure 5E**). NLR, which represents patient immune status, was also included in the study. Patients with a NLR over 5 had significantly lower Metabolites-Score than patients with a NLR below 5 (2.14 ± 1.88 vs. 3.56 ± 2.60, P=0.012, **Figure 5F**).

### 3.6 Metabolomic markers predicting prognosis of AFP negative HCC in liver transplantation

**Figure 6:**
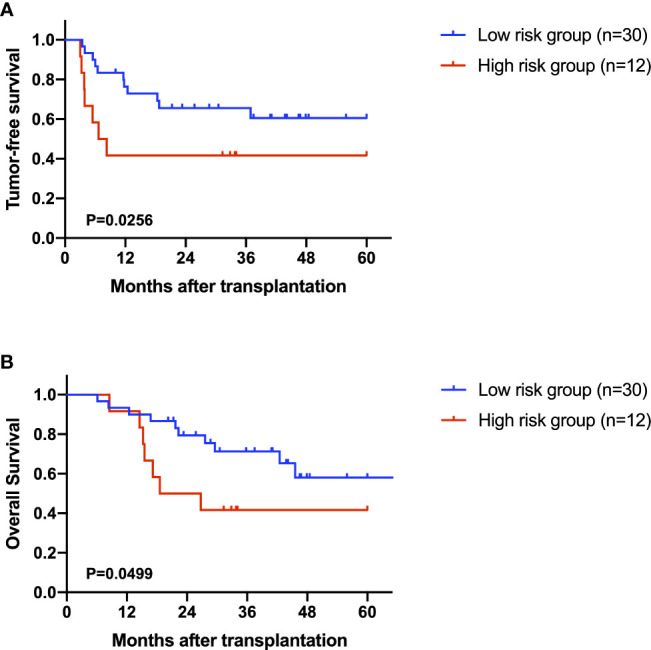
The role of MG(18:2/0:0/0:0) in the prediction of prognosis. **(A)** Kaplan-Miere plot of tumor-free survival in AFP negative HCC patients. **(B)** Kaplan-Miere plot of overall survival in AFP negative HCC patients.

After excluding the patients who died within two months, 42 AFP negative HCC patients were enrolled for prognostic analysis. 17 patients died during follow-up, with 1-, 3-, and 5-year overall survival (OS) rates of 92.9%, 62.8% and 52.2%, respectively. 18 patients were diagnosed with tumor recurrence during follow-up, with 1-, 3-, and 5-year tumor-free survival (TFS) rates of 66.5%, 58.9% and 54.7%, respectively. According to the univariable Cox regression analysis, MG(18:2/0:0/0:0) was the only metabolite having a moderate prediction capability for TFS (HR=1.160, 95%CI 1.012-1.330, P=0.033, **Table 3**). Based on the normalized peak intensity of MG(18:2/0:0/0:0), the patients were divided into low risk group (n=30) and high risk group (n=12). TFS and OS was significantly different between the two groups (P<0.05, **Figures 6A, B**), especially in early survival. We also validated the prognostic value of MG(18:2/0:0/0:0) in AFP positive HCC patients, but it showed no difference between low risk group (n=55) and high risk group (n=19, **Supplementary Figures S3A, B**).

**Table 3:** Univariate Cox regression analysis for predictive factors of tumor-free survival.

Ions	Metabolites	HR (95% CI)	P value
var690	PC(16:0/16:0)	0.977 (0.943-1.012)	0.190
var634	1-Oleoylglycerophosphoserine	0.909 (0.797-1.037)	0.156
var325	PC(18:2/18:2)	0.994 (0.979-1.010)	0.471
var810	PC(14:0/20:4)	1.067 (0.816-1.394)	0.637
var61	SM(d18:1/18:1)	0.965 (0.876-1.063)	0.465
var4	LysoPC(16:0)	1.003 (0.990-1.016)	0.681
var169	PC(22:6/18:2)	0.959 (0.817-1.125)	0.608
var265	PC(22:6/16:0)	1.005 (0.995-1.016)	0.329
var499	MG(18:2/0:0/0:0)	1.160 (1.012-1.330)	0.033
var312	3-Methoxybenzenepropanoic acid	1.031 (0.975-1.090)	0.290
var297	Chenodeoxycholic acid glycine conjugate	0.986 (0.971-1.002)	0.078
var380	LysoPC(17:0)	1.011 (0.779-1.312)	0.933
var905	PC(18:2/20:4)	1.028 (0.983-1.075)	0.227
var350	DG(9M5/9M5/0:0)	0.801 (0.480-1.335)	0.395
var898	3-Carboxy-4-methyl-5-propyl-2-furanpropionic acid	1.359 (0.827-2.232)	0.226

HR, hazard ratio; CI, confidence interval.

## 4 Discussion

Multiple studies have reported that AFP negative HCC patients were less likely to feature aggressive tumors and were more likely to have a favorable long-term survival when compared with AFP positive HCC patients. Discrimination of AFP negative HCC from LC patients by noninvasive methods is important for clinical practice, which would help patients to get timely and appropriate treatment. A number of serum biomarkers carrying diagnostic potential, like des-gamma-carboxyprothrombin (DCP), and lens culinaris agglutinin-reactive AFP (AFP-L3), have been identified as complements to AFP (15). Furthermore, Xu et al. reported that the combination of AFP-L3 and glypican-3 (GPC3) achieved high diagnostic accuracy for low-AFP HCC patients, because single detection with AFP-L3 may not be sensible and accurate (16, 17). Combination of Dickkopf proteins (DKK1) and AFP also increased the diagnostic yield than using either marker alone (18, 19). Studies are still being carried out for optimal biomarkers for AFP negative HCC. Metabolomics has always been a method exploring new diagnostic markers for various liver diseases. Here, we performed metabolomic profiling on the plasma of healthy volunteers and patients with LC or HCC to select novel biomarkers. Our results showed that the combination of metabolomic biomarkers could be applied to distinguish AFP negative HCC patients and predict their outcomes.

In this study, we identified 15 markers able to discriminate AFP negative HCC from both LC and HC patients. These markers are associated with glycerophospholipid metabolism. Alterations in glycerophospholipid metabolism was involved in the progression of different kinds of cancer including HCC (20–22). It is reported that highly proliferating cancer cells need to continually provide glycerophospholipids particularly for membrane production by fatty acids synthesis (23). On the other hand, among the 15 markers, 8 of them are also significantly altered between POS and LC group, which indicated that involved metabolomic changes were common in HCC pathologically. Thus, targeting this pathway might be a promising strategy for HCC treatment. For instance, Sorafenib, which is the most common drug for targeted therapy in HCC, could preferentially affect glycerophospholipid metabolism (24). We further performed Random forest analysis and Logistic regression analysis to construct the novel model. The three-marker model is accurate to distinguish AFP negative HCC patients from liver cirrhosis patients. By combining AFP with this model, we are able to achieve higher accuracy for diagnosis with an AUROC of 0.951.

Our three-marker model contains two kinds of phosphatidylcholine (PC) and one kind of sphingomyelin (SM). Many studies have reported their association with cancer and other disorders, which is known to play an important role in biological function including cell proliferation, migration and apoptosis (25, 26). A recent study found that the generation of PC is a notable lipid signature in proliferating hepatocytes, which also showed a positive correlation to hepatic carcinogenesis (27). Sphingomyelin synthase (SMS) is reported to play a critical role in sphingolipid metabolism which is involved in oncogenesis and sorafenib resistance (28), though the direct function of SM in HCC has not been clearly elucidated. Nevertheless, different types of PCs also have diverse functions. Some studies indicating that PC showed opposite function in tumor progression and hepatic carcinogenesis (29, 30). Our research also reflected this contrary phenomenon, that is, increased PC(16:0/16:0) showed lower risk of HCC, while increased PC(18:2/18:2) had a positive relationship to the risk of HCC. Subsequently, we further studied the correlation between the Metabolites-Score and clinical parameters. Our results found that patients in different groups divided by tumor parameters (including AFP level, tumor number and largest tumor size) have close Metabolites-Score, which indicated our model is applicable to all kinds of HCC patients. As for body nutrition parameters, overweight (BMI≥24kg/m^2^) and non-sarcopenic patients had relatively high Metabolites-Score, though without significant difference due to low sample size. Several studies reported that overweight and sarcopenic patients had distinctive lipidomic signatures like dysregulated SM and PC lipid species (31–33). Interestingly, our results found that NLR, an inflammatory marker, was significantly related to Metabolites-Score. NLR could partially represent the balance between pro-tumor inflammation and anti-tumor immune reaction (34). It is reported that PC-derived lipid mediators could bind to receptors presented in diverse immune cells, thus inhibiting the antitumor immunity and promoting immunoregulation (35). Therefore, metabolomics or lipidomics is promising to identify novel biomarkers to reflect body immune status and metabolic status concurrently.

Also, we found that MG(18:2/0:0/0:0) was associated with both OS and TFS in AFP negative patients, though it was not applicable for all HCC patients. This finding indicated that MG(18:2/0:0/0:0) was a prognostic biomarkers specially for AFP negative HCC. MG(18:2/0:0/0:0) belongs to monoglyceride family, which is more correctly known as a monoacylglycerol. Yang et al. reported that the overexpression of monoglyceride lipase (MGLL), an enzyme converting monoacylglycerol to free fatty acids and glycerol, could suppress the migration of HCC cells (36). Thus, monoacylglycerol might accumulate in patients with advanced HCC due to the deficit of MGLL.

Our study still has some limitations. Firstly, non-targeted metabolomics has disadvantages such as inaccurate identification of metabolites, difficult to detect low abundance metabolites and so on. For new model establishment, the differential metabolites were relatively scarce. In spite of those disadvantages, we still provided a perspective on metabolic markers for AFP negative HCC and identified several lipid metabolism-associated markers. Hence, targeted metabolomics like lipidomics could be performed accordingly in the future. Secondly, due to the severe burden of HCC in China, the recurrence rate was relatively high in this study for the attempts in liver transplantation beyond the Milan criteria. Thus, those identified metabolites and the nomogram might not be completely suitable for western patient cohort. Another issue is its feasibility in clinical practice, so further external validation should be performed in future studies.

In conclusion, metabolomics profiling successfully identified metabolic markers and novel diagnostic nomogram for AFP negative HCC. The pre-operative plasma metabolite level was also efficient in the prediction of recurrence risk in liver transplantation for AFP negative HCC.

## Data availability statement

The original contributions presented in the study are included in the article/**Supplementary Material**. Further inquiries can be directed to the corresponding author.

## Ethics statement

The studies involving human participants were reviewed and approved by the First Affiliated Hospital, Zhejiang University School of Medicine. The patients/participants provided their written informed consent to participate in this study.

## Author contributions

Study concept and design: XX, SZ and DL; Acquisition of data: ZL, HL and CH; Analysis and interpretation of data: ZL, HL, CH, MY and DL; Drafting of the manuscript: ZL, HL, HC and DL; Critical revision of the manuscript for Important intellectual content: XX, SZ and XW; Statistical analysis: ZL, XY, JZ, WS and ZH; Obtained funding: XX and DL; Administrative, technical, or material support: WS, ZH and LP; Study supervision: XX. All authors contributed to the article and approved the submitted version.
