# Supplementary material for: Metabolomic biomarkers for the diagnosis and post-transplant outcomes of AFP negative hepatocellular carcinoma
Source: Front Oncol. 2023 Feb 9;13:1072775. doi: 10.3389/fonc.2023.1072775 (PMC9947281; doi:10.3389/fonc.2023.1072775)
Supplement: Supplementary file 1 [file DataSheet_1.docx]

Supplementary Material

This file includes:

Supplementary Table S1-S4

Supplementary Figure S1-S3

Supplementary Table S1. Baseline characteristics of 122 HCC patients

| Characteristics | n/n |
| --- | --- |
| AFP level (≤400 ng/mL/ >400ng/mL) | 84/38 |
| Tumor number (single/multiple) | 51/71 |
| Tumor size (≤5 cm/>5 cm) | 79/43 |
| BMI (<24kg/m^2^/≥24kg/m^2^) | 82/40 |
| Sarcopenia (no/yes) | 95/27 |
| NLR (≤5/>5) | 96/26 |
| Post-transplant recurrence (no/yes) | 57/65 |

HCC, hepatocellular carcinoma; AFP, α-fetoprotein; BMI, body mass index; NLR: neutrophil-lymphocyte ratio.

Supplementary Table S2. Differential ions and referred information between LC and NEG group

| Ions | Metabolites | Retention time | m/z | Molecular ion |
| --- | --- | --- | --- | --- |
| var297 | Chenodeoxycholic acid glycine conjugate | 10.17 | 414.2995 | [M+H-2H_2_O]^+^ |
| var499 | MG(18:2/0:0/0:0) | 12.95 | 377.2668 | [M+Na]^+^ |
| var634 | 1-Oleoylglycerophosphoserine | 10.16 | 488.2698 | [M+H-2H_2_O]^+^ |
| var690 | PC(16:0/16:0) | 19.27 | 734.5661 | [M+H]^+^ |
| var350 | DG(9M5/9M5/0:0) | 16.93 | 673.5307 | [M+H]^+^ |
| var265 | PC(22:6/16:0) | 18.7 | 806.5713 | [M+H]^+^ |
| var61 | SM(d18:1/18:1) | 18.54 | 729.5894 | [M+H]^+^ |
| var380 | LysoPC(17:0) | 13.29 | 510.3567 | [M+H]^+^ |
| var4 | LysoPC(16:0) | 12.61 | 991.6735 | [2M+H]^+^ |
| var169 | PC(22:6/18:2) | 18.3 | 830.5698 | [M+H]^+^ |
| var325 | PC(18:2/18:2) | 18.35 | 782.5701 | [M+H]^+^ |
| var312 | 3-Methoxybenzenepropanoic acid | 8.87 | 181.0859 | [M+H]^+^ |
| var905 | PC(18:2/20:4) | 18.26 | 806.5652 | [M+H]^+^ |
| var898 | 3-Carboxy-4-methyl-5-propyl-2-furanpropionic acid | 8.86 | 223.0979 | [M+H-H_2_O]^+^ |
| var810 | PC(14:0/20:4) | 17.93 | 754.5398 | [M+H]^+^ |

MG, monoacylglyceride; PC, phosphatidylcholine; DG: diglyceride; 9M5, 9-(3-methyl-5-pentylfuran-2-yl)nonanoic acid; SM, sphingomyelin.

Supplementary Table S3. Logistic regression analysis for diagnosis of AFP negative HCC

| Ions | Metabolites | HR (95% CI) | P value |
| --- | --- | --- | --- |
| var690 | PC(16:0/16:0) | 0.932 (0.888-0.977) | 0.004 |
| var634 | 1-Oleoylglycerophosphoserine | 0.981 (0.787-1.222) | 0.933 |
| var325 | PC(18:2/18:2) | 1.038 (1.004-1.073) | 0.027 |
| var810 | PC(14:0/20:4) | 1.550 (0.783-3.070) | 0.208 |
| var61 | SM(18:1/18:1) | 1.340 (1.036-1.734) | 0.026 |
| var169 | PC(22:6/18:2) | 1.043 (0.615-1.768) | 0.877 |
| var265 | PC(22:6/16:0) | 0.990 (0.968-1.012) | 0.367 |

HCC, hepatocellular carcinoma; AFP, α-fetoprotein.

Supplementary Table S4. Diagnostic performance of AFP, three-marker model and combination model

| Model | AUROC | ROC Comparisons | | Parameters in Youden’s index of the model | | | |
| --- | --- | --- | --- | --- | --- | --- | --- |
|  |  | ΔAUROC | P value | Sensitivity | Specificity | PPV | NPV |
| AFP | 0.812 (0.716-0.909) | - | - | 0.91 | 0.6 | 0.917 | 0.577 |
| Three-marker model | 0.912 (0.857-0.967) | 0.1* | 0.13* | 0.713 | 0.92 | 0.978 | 0.397 |
| Combination model | 0.951 (0.917-0.986) | 0.139*  0.039^#^ | 0.014*  0.006^#^ | 0.861 | 0.92 | 0.981 | 0.575 |

AFP, α-fetoprotein; AUROC, area under the time-dependent receiver operating characteristic curve; PPV, positive predictive value; NPV, negative predictive value.

*: Comparing with AFP; ^#^: Comparing with three-marker model.


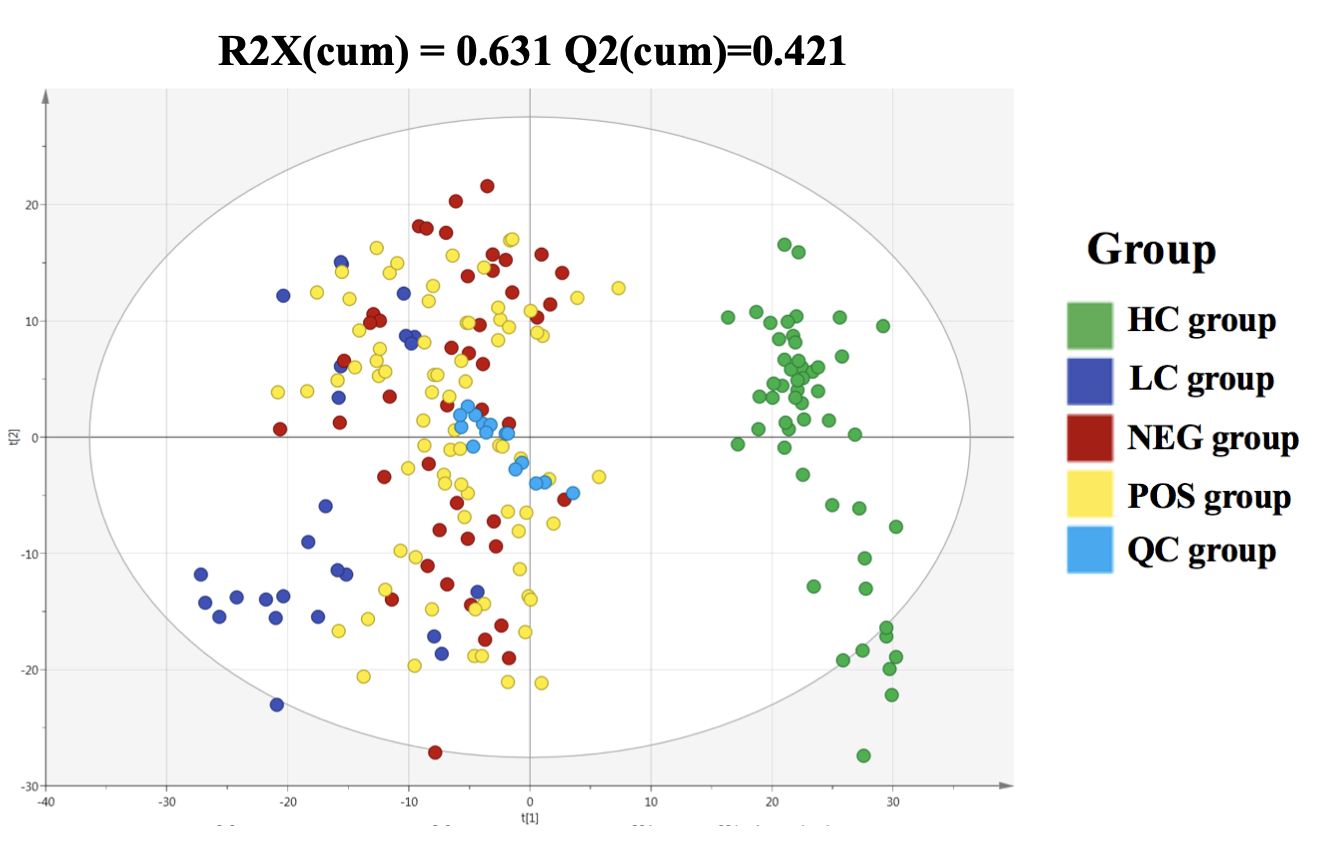


**Supplementary Figure S1. PCA score plot for samples of healthy volunteers, patients with liver diseases and quality controls.**


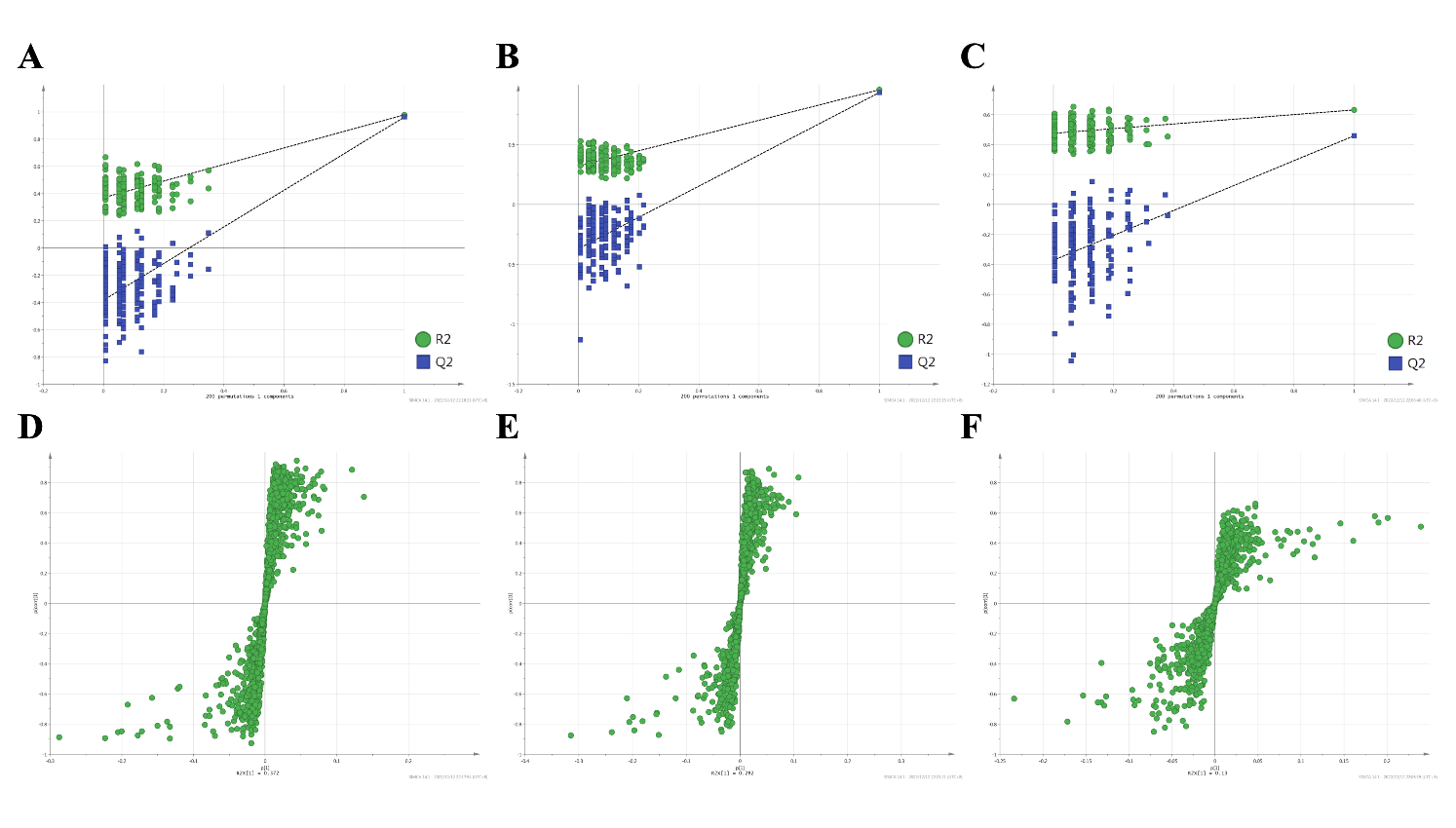


**Supplementary Figure S2. Validation plot and S plot of the OPLS-DA model.**

Validation plot of the OPLS-DA model of (A) HC vs. LC group, (B) HC vs. NEG group and (C) LC vs. NEG group, which were obtained from 200 permutation tests. S plot of the OPLS-DA model of (D) HC vs. LC group, (E) HC vs. NEG group and (F) LC vs. NEG group.


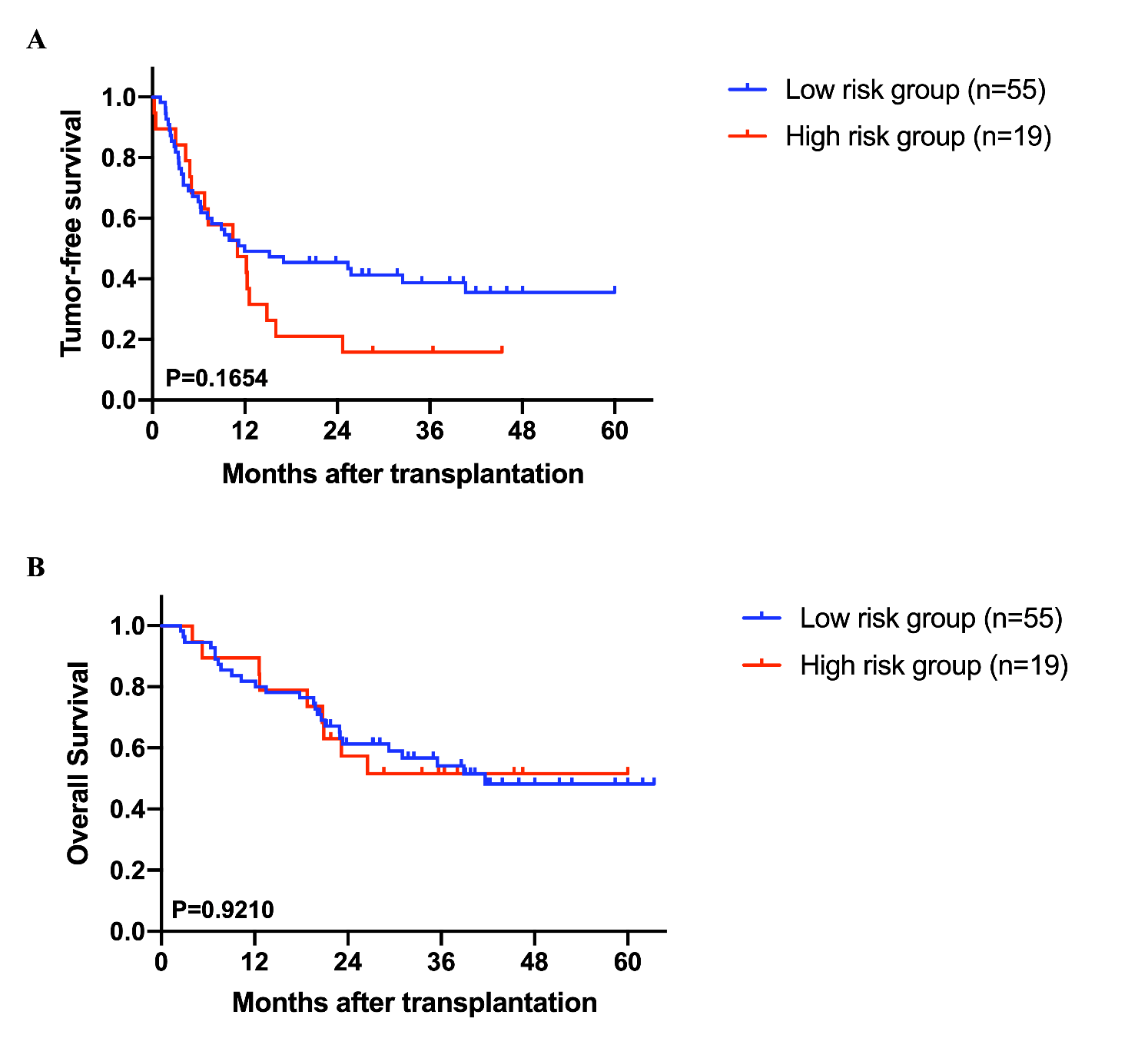


**Supplementary Figure S3. The role of MG(18:2/0:0/0:0) in the prediction of prognosis.**

(A) Kaplan-Miere plot of tumor-free survival in AFP positive HCC patients. (B) Kaplan-Miere plot of overall survival in AFP positive HCC patients.
